# What Drives People to Share Misinformation on Social Media during the COVID-19 Pandemic: A Stimulus-Organism-Response Perspective

**DOI:** 10.3390/ijerph191811752

**Published:** 2022-09-17

**Authors:** Manli Wu

**Affiliations:** School of Journalism and Information Communication, Huazhong University of Science and Technology, Wuhan 430074, China; mlwu@hust.edu.cn

**Keywords:** misinformation sharing, COVID-19, social media dependency, stimulus-organism-response

## Abstract

(1) Background: Misinformation is prevalent on social media in the age of COVID-19, exacerbating the threat of the pandemic. Uncovering the processes underlying people’s misinformation sharing using social media assists people to cope with misinformation during the COVID-19 pandemic. This study extends the stimulus-organism-response framework to examine how individuals’ social media dependency relates to their misinformation sharing behavior, with a focus on the underlying processes. (2) Methods: A total of 393 valid questionnaires were collected using a survey method to test the proposed research model. (3) Results: The results demonstrate that informational dependency and social dependency engender both positive and negative cognitive states, namely perceived information timeliness, perceived socialization and social overload, which then invoke positive as well as negative affect. What is more, the results show that both positive affect and negative affect can engender misinformation sharing. (4) Conclusions: Theoretically, this study uncovers the processes that lead to misinformation sharing on social media during the COVID-19 pandemic. Practically, this study provides actionable guidelines on how to manage social media usage and social media content to cope with misinformation sharing during the pandemic.

## 1. Introduction

The popularity of social media has expedited the dissemination of information. However, owing to the large volume of information with unidentified sources, the problem of misinformation on social media becomes prominent [1]. For instance, Sina Weibo’s latest report revealed that more than sixty thousand pieces of misinformation were processed by the website in 2021. According to the World Economic Forum, online misinformation is a growing global threat to humanity [2,3]. Notably, concerns on the sharing of misinformation keep arising during the COVID-19 pandemic, causing the emergence of an infodemic [4,5]. Misinformation regarding the imaginable health impacts of COVID-19 and its terrible consequences circulated on different social media platforms, causing great anxiety and panic [5]. It is believed that the proliferation of misinformation increases the threat of COVID-19 [6]. As such, the World Health Organization has labelled tackling the infodemic as being as critical as the pandemic. The prevalence of misinformation associated with its damaging consequences motivates us to uncover the processes underlying misinformation diffusion.

The existing literature has made efforts to understand the diffusion of misinformation. Some scholars attempted to uncover the processes underlying misinformation diffusion. For instance, Hui et al. [7] adopted the epidemic-like model to examine the spread process of misinformation on social media. To understand the process of online rumor spreading, Wang et al. [8] built on the SIR epidemic model to simulate the rumor propagation-reversal process. Other researchers also explored the propagation patterns of misinformation on social media platforms [9,10]. These studies were carried out from a group view, failing to take individual behavior into consideration. Another stream of research investigated the reasons behind individuals’ misinformation sharing. For instance, Kim and Dennis [11] examined how the presentation formats and source ratings of fake news affected social media users’ believability and sharing. Freiling et al. [5] investigated the effect of political viewpoints on social media users’ misinformation believability and sharing. Other studies also revealed that online information trust, social comparison, information overload, social media fatigue, and information literacy were predictors of individuals’ misinformation sharing [2,4,12,13,14]. Prior studies tended to regard people as rational decision-makers and ignored that sharing was not entirely rational and might be emotionally driven. Moreover, little work has been carried out to explain individuals’ misinformation sharing by simultaneously considering its process as well as the reasons behind it. Although a group view is insightful in depicting the diffusion process, the wide spread of misinformation lies in the engagement of regular users, and individuals’ psychological processes play an important part in misinformation diffusion. Hence, this study integrates individuals’ psychological processes to explore why people share misinformation on social media.

Although a number of studies have discussed that social media attributes amplify the issue of misinformation sharing, these studies tend to regard social media as the research context rather than the focal artifact of the investigation, failing to incorporate its attributes into their theoretical frameworks [2,6,15]. While social media use has been conceptually discussed in relevant studies, it should be noted that social media are not monolithic and they can be used in various ways [16]. As such, there is a need to specify the distinct contextualization of misinformation sharing and examine the roles of different social media use in stimulating individuals’ misinformation sharing. Individuals are influenced by social media because they rely on it to meet their goals [17]; therefore, this study uses social media dependency to reflect individuals’ dependency on social media. Considering that the attributes of social media entail people to depend on it for different purposes, this study thus distinguishes types of social media dependency and examines how these dependencies affect individuals’ misinformation sharing.

Motivated by the above research gaps, this study aims to answer the research question: *How do social media dependencies affect individuals’ misinformation sharing on social media platforms*? To address this question, the present study draws on the framework of stimulus-organism-response (S-O-R) to develop an integrated theoretical model, which seeks to elaborate on the development of misinformation sharing with individuals’ social media dependencies. Specifically, social media dependencies are conjectured as stimuli that could induce individuals to experience certain psychological processes, which in turn, lead to their misinformation sharing behavior. A survey method was employed to collect the data, and a total of 393 valid responses were obtained to test the proposed theoretical model.

This study is expected to have theoretical and practical implications. In theoretical terms, this study adds knowledge to the literature by extending the S-O-R framework to misinformation sharing research and building a holistic understanding of the process of misinformation sharing. In practical terms, by uncovering the process underlying the development of misinformation sharing, this study provides actionable guidelines on how to manage social media usage and social media content to cope with misinformation sharing in the age of COVID-19.

## 2. Theoretical Background

### 2.1. Misinformation Sharing on Social Media

Misinformation is conceptualized as claims that are not supported by clear scientific evidence [6]. The features of social media exacerbate the spread of misinformation. First, a mixture of information from multiple sources can be found on social media [11], while a rigorous quality control mechanism is lacking [15]. Second, the volume of information is quite large and the content is fragmented, which poses challenges to separating true information from false information [1]. Third, the social atmosphere created on social media enables people to engage in real-time information exchange and share information without deliberative thinking, which increases the possibility to diffuse misinformation [18].

A great deal of attention has been paid to explaining people’s misinformation sharing on social media in recent years. Prior studies have discussed a series of predictors, such as information believability [19], online trust [14], information literacy [13], and self-efficacy [20]. Researchers in these studies were inclined to conclude that individuals share misinformation because they lack enough rational thinking, and they attached less importance to the role of impulsive factors such as individuals’ affective states. Individuals’ behavior is driven by two mechanisms: one is an inhibitory, controlled, and reflective mechanism, and the other is an automatic, impulsive and reflexive mechanism [21]. The former supposes that behavior is determined by attitude and intention; The latter regards that behavior is driven by emotion rather than deliberation, and is a result of impulsion and unconsciousness. During the COVID-19 pandemic, people have been emotionally overwhelmed and they may disseminate information as an outlet of emotion [2]. Such emotional states can be referred to as affect. Prior studies have adopted rational choice theory, the health belief model, protection motivation theory, and so forth to explain users’ online behavior during the pandemic [2,14,22]. However, these theories treat behaviors as rational, failing to develop an in-depth understanding of behaviors that are emotionally driven. Therefore, this study regards affect as an important psychological process and examines its role in the development of misinformation sharing.

### 2.2. The Framework of Stimulus-Organism-Response

The S-O-R framework underlines the importance of individuals’ psychological processes in response to external stimuli [23]. The basic rationale of this framework is that external stimuli lead to internal reactions in individuals (i.e., organisms), which in turn, shape their behavior (i.e., responses) [24]. In light of S-O-R [24,25], stimuli are environmental cues that individuals confront in a certain situation, organisms are individuals’ internal cognitive and affective states, and responses refer to individuals’ behavioral outcomes in response to particular stimuli.

The S-O-R framework has been widely used to predict online behavior such as social media usage [26], purchase decisions [27], and game playing [23]. More recently, it has been employed to explain individuals’ online behavior in the face of the COVID-19 pandemic. For instance, guided by the S-O-R model, Laato et al. [22] regarded online information as environmental stimuli and examined how such stimuli resulted in self-isolation intention and unusual purchasing. Luo et al. [20] adopted the paradigm of S-O-R to investigate how stimuli from peer influence affected individuals’ rumor sharing. Song et al. [28] built their work on the S-O-R model to illustrate how external stimuli, i.e., information overload and the threat of COVID-19, led to online information avoidance. Given the importance of social media in triggering online behaviors, these studies only regarded it as the background context and did not conceptualize it as an effective stimulus. Originating from the media dependency theory, social media dependency describes that individuals are influenced by social media because they depend on it to achieve certain goals [17]. Specifically, individuals who depend on social media for information exchange and relationship development are more likely to engage in information sharing. Hence, social media dependency can serve as an environmental stimulus that motivates misinformation sharing. This study conceptualized social media dependency as a stimulus and adopted the S-O-R framework to examine how it relates to misinformation sharing in the age of COVID-19. First, this framework divides stages of behavior formation and sheds light on how external stimuli affect individuals’ internal states and ultimately result in certain behavioral responses, which helps us uncover the mechanisms underlying the development of misinformation sharing. Second, by highlighting individuals’ psychological processes in reaction to environmental stimuli, this framework goes beyond rationality assumption and explains individuals’ information dissemination behavior in the context of COVID-19, where behavior is usually driven by emotions and is not entirely rational. Third, the organism component in the S-O-R model emphasizes both positive and negative orientations toward specific stimuli [29], lending itself to be effective in understanding individuals’ different psychological reactions in response to environmental stimuli from social media usage.

### 2.3. Stimuli: Social Media Dependencies

People usually rely on social media to keep them informed and help them to stay in touch with others during the COVID-19 pandemic [22], and social media dependency can cause changes in their psychological states as well as behavior. The present study thus regards social media dependency as an external input that stimulates internal reactions, which further induces the sharing of misinformation.

The media system dependency theory views media as an information system and proposes that individuals are affected by social media in the process of usage [30]. According to this theory, there are six subsets regarding social media dependency, namely personal understanding, solitary play, personal action orientation, interaction play, social understanding, and social action orientation [30]. The above subsets of social media dependency reflect that people use social media for either personal purposes or social purposes. Considering that individuals depend on social media to fulfill their information needs and social needs in the age of COVID-19 [22], this study identifies informational dependency and social dependency as the representation of social media use, and examines how people’s different dependencies on social media affect their misinformation sharing. Among the two dependencies, informational dependency refers to people’s dependency on social media to access to or obtain information to satisfy their informational needs [30]; social dependency is termed as people’s dependency on social media to engage in social interaction to fulfill their social needs [31].

### 2.4. Organisms: Cognitive and Affective States

The organisms are reflected by individuals’ cognitive and affective states [24]. Cognitive states refer to the cognitive processes regarding the acquisition and processing of information, and affective states represent emotional reactions in the face of certain stimuli [32]. Prior studies tend to use either cognitive states or affective states as the representation of the organism, failing to clarifying the relationship between these two states [22,23]. It is commonly agreed that individuals’ affective assessment depends on their cognitive evaluation of specific objects [33]. The argument indicates that cognitive components are correlated with affective components, with cognition being the predictor of affect. When studying misinformation sharing, researchers have examined both cognitive components and affective components as antecedents [2,12]. However, the interrelationship between cognitive and affective components are not clarified. Therefore, by extending the S-O-R framework, this study goes a step further and uncovers the impacts of cognitive states on affective states.

Social media is a double-edged sword that can generate both positive and negative outcomes [34,35]. For instance, individuals are used to depending on social media to obtain real-time information; meanwhile, the abundance of information may lead to information overload [4,12]. Individuals rely on social media to maintain social contact [36]; in the meantime, they may also experience social overload as they have many social interactions to engage in [37]. Therefore, social media dependency can lead to positive as well as negative cognitive and affective states. Combining the two types of social media dependency, this study conceptualizes perceived information timeliness, perceived information overload, perceived socialization, and perceived social overload as positive and negative cognitive states engendered by social media dependencies. Positive and negative affect may be evoked by individuals’ different cognitions [33]. As such, this study conceptualizes positive affect and negative affect as affective states, and examines how individuals’ cognitive states relate to their affective state.

As is specified in the framework of S-O-R, organisms mediate the relationship between stimuli and responses [25]. This study labels social media dependency as the stimulus and misinformation sharing behavior as the response. In summary, this study attempts to investigate how environmental stimuli (social media dependencies) cause changes in individual organisms (cognitive and affective states), which ultimately leads to a response (misinformation sharing behavior).

## 3. Research Model and Hypotheses

**Figure 1:**
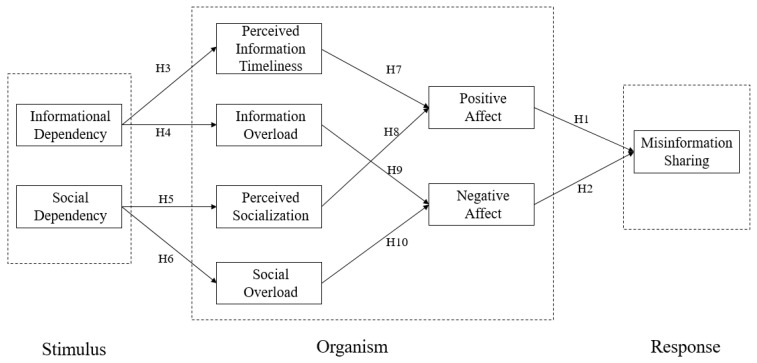
Research model.

This paper has developed a research model to illustrate individuals’ misinformation sharing behavior on social media during the COVID-19 pandemic. As depicted in Figure 1, individuals’ social media dependency causes changes in cognitive states, and cognitive states relate to affective states, which then exert effects on misinformation sharing.

### 3.1. The Role of Affect

Affect refers to the affective states of individuals, and it can be either positive or negative [38]. In the current study, positive affect can be represented by individuals’ feelings of desire, relaxation, and happiness, and negative affect involves the feelings of fear, anxiety, anger, and shock [39,40]. One usually acts online as an attempt to strengthen positive affect [41]. For instance, Twitter users are more willing to disseminate positive events [42]; Microblog users with positive affect are more willing to conduct real-time status updates [43]. Considering the situation of the pandemic, individuals usually gain positive affective states as a result of hopeful information as well as social companionship, which encourages them to share real-time information within their social networks. Hence, individuals with positive affect are likely to share information without verification, which may lead to misinformation sharing.

Negative affect may also exert an impact on misinformation sharing. People’s misinformation sharing behavior is mainly driven by their perceptions of information importance [44]. The feature of importance is strongly correlated with negative affect, such as anxiety [18]. In the age of COVID-19, the experience of negative affect strengthens people’s perceived importance of information, which in turn propels people to disseminate information without spending time conducting verification. In addition, negative affect can be a key predictor of misinformation sharing, since the sharing behavior can be conceptualized as an outlet of emotional pressure [18]. From this perspective, this study postulates that individuals’ misinformation sharing behavior will increase when the individual experiences strong positive or negative affect.

**H1:**  *Positive affect has a positive relationship with individuals’ misinformation sharing*.

**H2:**  *Negative affect has a positive relationship with individuals’ misinformation sharing*.

### 3.2. The Role of Social Media Dependency

People use social media for different purposes. Since social media can function as a useful means for information transmission, people depend on it for information acquisition [31]. For instance, a recent study revealed that, among all information sources, most adolescents regarded social media as their primary source of information [45]. Social media platforms, such as WeChat and Microblog, allow real-time information updates and transmission, which enables people to gain timely information. During a pandemic, people are in urgent need of information since the situation involves uncertainty and ambiguity [46]. As such, many people turn to social media to keep themselves informed, and follow the updates on the platform [2], which allows them to gain timely information. Information timeliness deals with whether the information is timely and current [47]. The present study therefore assumes that individuals’ informational dependency may affect their perceived information timeliness.

**H3:**  *Informational dependency on social media has a positive relationship with individuals’ perceived information timeliness*.

People nowadays are used to checking social media compulsively, leading to a wide range of information consumption [30,48]. Nevertheless, the abundance of information encountered may also result in information overload. Information overload describes the situations where the information on social media exceeds an individuals’ cognitive capacity [28,49]. During the pandemic, the information volume has been quite large and the information content has been fragmented, which poses challenges for people to deal with [1]. As social media exacerbates the diffusion of information, more people rely on the platform to gain information and they may experience more information overload.

**H4:**  *Informational dependency on social media has a positive relationship with individuals’ perceived information overload*.

Besides obtaining information, people also depend on social media to maintain social connections and pursue socialization [30]. Socialization is defined as the degree to which people use social media for satisfying social needs [50] and developing social capital [51]. People with high social dependency will be more motivated to engage in social interactions [52]. During the pandemic, the rule of proper social distancing has motivated people to depend on social media to keep in touch with each other [2]. By socially depending relationships on social media, people engage in social interactions to get social support and show concern for others [51]. Thus, once people are dependent on social media for social purposes, they resort to it to engage in social interactions and maintain social relationships, which increases their socialization perceptions.

**H5:**  *Social dependency on social media has a positive relationship with individuals’ perceived socialization*.

As many people rely on social media for social connections by exchanging information and engaging in social communication [31], they need to handle a large volume of messages and communication requests simultaneously, which may pose a social burden on them [49]. For instance, the uncertainty or fear associated with the pandemic has forced people to engage in social activities to adapt to the situation, causing them to feel overwhelmed. Moreover, social dependent users are usually well-embedded in their social networks. In this vein, they may have many social affairs to process and they are expected to offer social support for others [37], which can lead to social overload if it is not handled properly [49]. In summary, social media users who have high social dependency have to deal with too many social interactions, which occupies too much time and can engender social overload.

**H6:**  *Social dependency on social media has a positive relationship with individuals’ perceived social overload*.

### 3.3. The Antecedents of Positive Affect

In this section, the impacts of perceived information timeliness and perceived socialization on individuals’ positive affect are investigated. As a growing number of people use social media to seek information, the value of information timeliness is emphasized [23]. Information timeliness can affect users’ affect in two ways. First, during the pandemic, timely information would help users develop a good understanding of the current situation and take the necessary measures to cope with it. The timelier the information, the higher the perceived information usefulness and the higher the satisfaction [47]. Second, as people usually have a strong desire for real-time information, especially during the COVID-19 pandemic, obtaining timely information can satisfy their cognitive gratifications, which are expected to exert a positive effect on their positive affect. Therefore, this study assumes a positive relationship between information timeliness and positive affect.

A high level of socialization manifests that people do well in developing social relationships and improving social capital [51]. Socialization may result in positive affect in two aspects. On one hand, people do not merely engage in social interactions for instrumental purposes, they also have social goals [31]. Hence, high socialization can help them realize their goals and ultimately gain positive affect. On the other hand, people can develop interpersonal trust and the sense of belonging via socialization, which creates an atmosphere for people to express themselves freely and further fosters positive affect such as comfort and enjoyment [53]. In addition, it is noted in the affective social exchange theory that interpersonal communication and social interactions exert positive effects on affective outcomes [54]. Past research also supports that social interactions exert positive effects on individuals’ positive affect [55]. During the COVID-19 pandemic, socialization has empowered people to alleviate fear and gain comfort. Therefore, this study assumes that socialization exerts a positive impact on positive affect.

**H7:**  *Individuals’ perceived information timeliness has a positive relationship with their positive affect*.

**H8:**  *Individuals’ perceived socialization has a positive relationship with their positive affect*.

### 3.4. The Antecedents of Negative Affect

In this section, the impacts of information overload and social overload on individuals’ negative affect are examined. Individuals experience information overload when the volume of information they confront on social media exceeds what they are able to process [28,56]. Since the COVID-19 pandemic started, an increasing amount of relevant information in the form of text, photographs and video is posted on social media. The sheer volume of information makes it a challenge for people’s limited cognitive ability to deal with, which may cause people to experience the feeling of psychological strain [49,57]. In addition, the abundance of misinformation regarding the severity of the pandemic as well as the negative health impacts disseminated on various social media platforms, results in negative affective states such as distress and anxiety [5,22]. As such, information overload is postulated to exert a positive impact on individuals’ negative affect.

Social overload occurs when social media users have plenty of social requests to process [57]. Once social requests are beyond an individual’s processing ability, they will become overwhelmed and experience a feeling of losing control, which are strong predictors of negative affect [49,56]. The negative effects of social overload will be more evident in the age of COVID-19, where individuals are more likely to experience health anxiety, and they utilize social interaction as an outlet of the negative affect. In this sense, the social contagion of negative affect can be accelerated by social overload. Therefore, social overload is assumed to have a positive influence on negative affect.

**H9:**  *Perceived information overload has a positive relationship with individuals’ negative affect*.

**H10:**  *Perceived social overload has a positive relationship with individuals’ negative affect*.

## 4. Research Method

### 4.1. Data Collection and Sample

A survey method was utilized for collecting data. Prior to data collection, the developed questionnaire was sent to three postgraduate students and two assistant professors, and they were asked to make comments on the questionnaire. According to their comments, the clarity of the questions was refined. Therefore, the content validity of the questionnaire was guaranteed. Then, 65 active users of social media were invited to participate in a pilot survey, and the results validated the revised questionnaire.

The formal data collection was carried out in January 2021, during which time, there were COVID-19 outbreaks in some areas of mainland China, causing restrictions on citizens’ mobility. During this period, various types of misinformation regarding the COVID-19 pandemic were disseminated on social media platforms. At the beginning of the survey, we described the research context and gave examples of misinformation in order to help subjects develop a good understanding of the research situation. An example of misinformation is that Shuanghuanglian can cure COVID-19, and another example is that taking VC can prevent infection from COVID-19. Subjects were required to honestly report their recent feelings and behaviors during the COVID-19 pandemic.

The questionnaire was distributed on an online survey platform (www.wjx.cn, accessed on 15 January 2021) in China. By virtue of the sampling service provided by the platform, this study was able to select subjects randomly and, thereby, the representativeness of the sample was ensured. Screening questions were designed to guarantee the effectiveness of the responses. The subjects were active social media users and they were required to report the social media platform they used most and the frequency of their usage. All subjects were above 18 years of age. A total of 426 responses were obtained. After discarding 33 invalid responses, 393 valid responses remained. It has been recommended that 15 to 20 observations per variable are appropriate in the structural model [58]. Since there were nine variables in this study, a minimum of 180 responses was acceptable. Hence, 393 responses were enough in this study. All subjects were Chinese. Table 1 depicts the basic information of respondents.

### 4.2. Instrument Development

The instrument was developed by referring to measures in the existing literature. Measures for social dependency and informational dependency were adapted from Lee and Choi [30]. Measures for social overload and information overload were formed by adapting from Laato et al. [2] and Fu et al. [57], respectively. The work of Cheung et al. [47] was referred to in order to measure the perceived information timeliness. Perceived socialization was measured by referring to items from Kizgin et al. [51]. To measure positive affect and negative affect, this study referred to Moroń and Biolik-Moroń [39] and Yeung and Fung [40]. The two studies were both concerned with individuals’ emotional responses to the pandemic. This study adapted items from Apuke and Omar [36] and Laato et al. [2] to measure misinformation sharing. A seven-point Likert scale was adopted to measure the above items. Appendix A(Table A1) lists the measurement instruments for each construct.

### 4.3. Control Variables

Control variables are those factors that are not of interest in this study, but they could influence the outcomes. In order to exclude the confounding effects of other factors, this study included several factors as control variables, namely gender, age, education, and social media usage experience. We controlled for gender, age, and education because these factors were found to play important roles in individuals’ information sharing [6,15]. Since technology usage experience may affect individuals’ information sharing behavior [59], social media usage experience was also included as a control variable.

## 5. Data Analysis and Results

Misinformation sharing on social media has not been fully explored and a relevant theory needs to be established. Hence, this study adopted SmartPLS to conduct the data analysis since it is effective for exploratory studies [60].

### 5.1. Measurement Model Test

This study tested the measurement model by computing convergent and discriminant validity. Convergent validity can be assessed by examining composite reliability (CR), average variance extracted (AVE), and the standardized factor loading. As shown in Table 2, the CRs for all constructs were higher than the recommended benchmark of 0.7, and the AVEs for all constructs exceeded the suggested value of 0.5 [61]. This study then checked item loadings for each construct. It was revealed that all item loadings were above the threshold value of 0.7. Therefore, the requirements of convergent validity were satisfied. Discriminant validity can be evaluated by comparing the square root of the AVE of a construct with the inter-construct correlation coefficients [61]. The results in Table 3 validated the discriminant validity by showing that all square roots of the AVE were larger than any correlation coefficient between constructs.

This study checked multicollinearity by calculating the variance inflation factor (VIF) of the related variables. Multicollinearity was regarded as a concern if the VIF value was above 10 [58]. The results showed that value of VIF for each variable was between 1.269 and 2.039. Therefore, multicollinearity is not likely to be an issue.

This study also tested the common method variance (CMV). First, it is suggested that the CMV may lead to high correlations between constructs (r > 0.90). As revealed in Table 3, the highest correlation between constructs was 0.64, suggesting that CMV did not affect the results of this research. Second, the Harman’s single-factor test was employed [62]. The results showed that the highest variance explained by a single factor was only 30.87%, indicating the absence of CMV. Third, a partial correlation method was utilized to test CMV [62]. Specifically, this study added a control variable, which was the highest factor from the principal component factor analysis, in the research model. The findings revealed that the inclusion of this control variable failed to significantly improve the explained variance of our research model. Therefore, CMV is not likely to be a major concern in this research.

### 5.2. Structural Model Test

**Figure 2:**
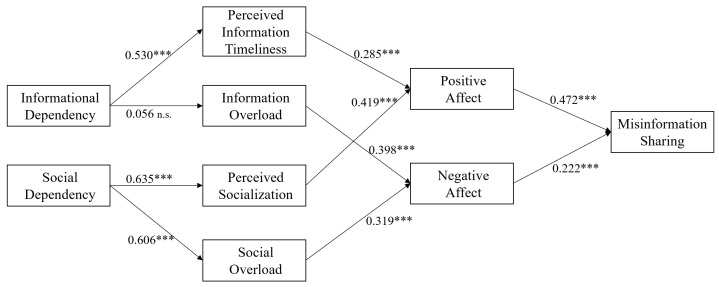
Results of data analysis. ***: *p* < 0.001; n.s.: not significant.

To test the proposed hypotheses, the path coefficients and corresponding *p*-values of the paths in the structural model were calculated. The results are depicted in Figure 2. The effects of control variables were examined. Age (β = 0.069, *p* > 0.05), gender (β = −0.005, *p* > 0.05), education (β = 0.052, *p* > 0.05), and social media usage experience (β = 0.041, *p* > 0.05) were all found to have no significant correlation with individuals’ misinformation sharing. Taken as a whole, the model explained 35.6% of the variance in misinformation sharing.

H1 and H2 concentrated on uncovering the effects of positive affect and negative affect on misinformation sharing. The results demonstrated that positive affect (β = 0.472, *p* < 0.001) and negative affect (β = 0.222, *p* < 0.001) exerted positive impacts on misinformation sharing, indicating that individuals would be more likely to share misinformation when they experienced strong affect. Hence, the results supported H1 and H2.

This study further tested H3, H4, H5, and H6 by examining the impacts of informational dependency and social dependency. Consistent with the argument in H3, the relationship between informational dependency and perceived information timeliness was positive as well as significant (β = 0.530, *p* < 0.001). As a result, H3 was supported. Nevertheless, the results failed to find a significant effect of informational dependency on information overload (β = 0.056, *p* > 0.05), indicating that H4 was not supported. Furthermore, the results showed that social dependency was positively associated with perceived socialization (β = 0.635, *p* < 0.001) and social overload (β = 0.606, *p* < 0.001), lending support to H5 and H6.

H7, H8, H9, and H10 analyzed whether individuals’ cognitive states affected their affective states, which are represented by positive affect and negative affect. The results revealed that the impacts of perceived information timeliness (β = 0.285, *p* < 0.001) and perceived socialization (β = 0.419, *p* < 0.001) on positive affect were positive and significant. Therefore, H7 and H8 were supported. The effects of information overload (β = 0.398, *p* < 0.001) and social overload (β = 0.319, *p* < 0.001) on negative affect were also found to be positive and significant. Thus, H9 and H10 were supported.

This study built on the S-O-R framework to examine the processes underlying misinformation sharing. Notably, this study also extends this framework to explore the impacts of cognitive states on affective states, with an emphasis on the mediation effects of affective states. Extensive research using S-O-R has validated the mediation effects of organisms; however, the mediating role of affect has not been clarified. To examine the potential effects of positive affect and negative affect, this study conducted a mediation effects test [63]. The results of the mediation effects are shown in Table 4. The direct impacts of perceived information timeliness (β = 0.147, *p* < 0.05) and perceived socialization (β = 0.219, *p* < 0.01) on misinformation sharing were significant. When positive affect was included, the effect of perceived information timeliness became insignificant (β = 0.084, *p* > 0.05), and the effect of perceived socialization decreased but was still significant (β = 0.140, *p* < 0.05). Therefore, positive affect fully mediated the effect of perceived information timeliness, and partially mediated the effect of perceived socialization on misinformation sharing. In addition, the direct impact of social overload on misinformation sharing was significant (β = 0.401, *p* < 0.001), while the effect decreased when negative affect was included (β = 0.304, *p* < 0.001). Therefore, the effect of social overload on misinformation sharing was partially mediated by negative affect. However, the effect of information overload on misinformation sharing was not significant (β = 0.003, *p* > 0.05), providing no support for the mediation effect of negative affect between information overload and misinformation sharing.

## 6. Discussion

### 6.1. Key Findings

By examining the antecedents of misinformation sharing and the underlying mechanisms, this study generates some interesting findings. First, the findings suggest that both positive affect and negative affect are positively correlated with misinformation sharing. The findings highlight the importance of affect in inducing misinformation sharing. In addition, the results show that positive affect (β = 0.472) exerts a higher impact on misinformation sharing than negative affect (β = 0.222). This finding can be explained by the situation under investigation. During the COVID-19 pandemic, a lot of misinformation has been concerned with the prevention of and treatments for COVID-19, which are favored by people. Since such information is accompanied with desire and relaxation, people are more likely to share it.

Second, the results demonstrate that individuals’ dependency on social media may lead to both positive and negative outcomes. Specifically, social dependency is found to be positively related to perceived socialization and social overload. Informational dependency exerts a positive impact on perceived information timeliness. The findings echo the viewpoint that social media usage is not monolithically good or bad, and social media use can be simultaneously favorable and unfavorable [34,35].

Third, there is one unexpected result. The findings failed to support the relationship between informational dependency and information overload. One possible explanation is that individuals have a high demand for information regarding COVID-19 due to the ambiguity of the situation [46]. The preference for information allows people to engage in more information consumption and deal with more information, which lowers people’s perception of information overload. Therefore, social media users’ informational dependency may not lead to information overload.

### 6.2. Theoretical and Practical Implications

Although misinformation sharing on social media has drawn considerable attention from practitioners for its side effects, theoretical understanding on its antecedents and the underlying processes is relatively rare. To address this research gap, this study examines the enacting factors associated with misinformation sharing and the underlying processes.

This study has several implications for research. First and foremost, this study contributes to research on misinformation sharing by verifying that both positive affect and negative affect are strong predictors of social media users’ misinformation sharing behavior. The relevant literature has pervasively examined sharing behavior based on a reflective mechanism, and regarded attitude and intention as the main predictors [21]. Taking the situation of the COVID-19 pandemic into consideration, this study goes a step further and proposes that individuals’ misinformation sharing behavior is not entirely rational and may be driven by their affective states. Following this line of reasoning, this study validates the role of affect in predicting misinformation sharing.

Second, this study suggests that individuals’ social media dependency leads to both desirable and undesirable outcomes. On one hand, individuals’ social media dependency allows them to experience information timeliness and socialization. On the other hand, individuals are also burdened with multiple pieces of information and ceaseless social interactions. Prior studies regarding the role of social media use have generated inconsistent findings, and existing attempts tend to focus on either positive or negative outcomes [34,35]. This study extends the current understanding on the consequences of social media usage by simultaneously considering positive and negative impacts in relation to social media dependency.

Last but not least, this study contributes to the literature by extending the S-O-R framework to offer a comprehensive perspective to illustrate how social media dependency affects misinformation sharing behavior. Regarding the S-O-R framework, prior studies have tended to use either cognitive states or affective states as the organism [22,23]. This study includes both cognitive states and affective states, and further tests their interrelationships, thus extending the application of the S-O-R framework in explaining the psychological processes underlying misinformation sharing.

This study also yields implications for practice. First, the results demonstrate that social media dependency will lead to both favorable and unfavorable affective states, which exert important impacts on misinformation sharing. As social media has become a popular platform for social interaction and information sharing [51], it is critical for managers and service providers to develop effective strategies to reduce users’ misinformation sharing behavior during the COVID-19 pandemic [12]. Our findings further suggest that strong affect increases individuals’ likelihood to share misinformation. Since misinformation is posted to attract attention, it usually contains emotional content that is appealing to readers [64]. For practitioners who take charge of social media content, the findings highlight that they should strengthen the content review mechanism with an emphasis on content with high emotional arousal.

Second, empirical findings highlight that social media use can influence individuals’ cognition, affect, and behavior [12,31]. Specifically, this study reveals that although social media dependency may provide a way of gaining timely information and maintaining social interaction, it can also cause overload. It should be noted that, while social media can bring a favorable experience, it can also lead to negative consequences, which need to be highlighted to social media users. As prior studies have indicated that excessive social media use may generate unfavorable cognitive and affective states [49,56], it is important for social media users to manage their media use behavior and reduce overuse on social media. In addition, our results show that emotional responses resulting from social media dependency may cause misinformation sharing. The findings reveal that social media users should regulate their emotion and avoid emotional bias when encountering information with a high emotional arousal.

### 6.3. Limitations and Directions for Future Research

There are several limitations that call for future research. First, this study utilizes cross-sectional data to validate the research model. Although such data can test the relationships between these constructs, they are limited in examining causality effects [65]. Since the research model is concerned with changes in cognition and affect, future research could consider using longitudinal data to capture the dynamics of the research model and examine the causal relationships between the constructs. In addition, since experiments are superior in testing causal relationships, a field experiment could be conducted to examine how individuals’ social media usage influences their cognitive and affective states, as well as their misinformation sharing behavior. Moreover, due to the pervasiveness of misinformation in social media, future research could also crawl social media data to analyze users’ sharing behavior.

Second, this study mainly examines how social media dependency relates to the development of misinformation sharing, and the model explains 35.6% variance of misinformation sharing. As proposed in a prior study, understanding individuals’ misinformation sharing behavior should consider technological, political, and societal factors [66]. Future research may wish to take more factors into account. For instance, besides technological factors, researchers can also explore how particular political and societal factors relate to misinformation sharing.

Third, subjects in this study are social media users of mainland China, and the findings may reflect their misinformation sharing behavior during the COVID-19 pandemic. However, whether the findings are applicable to other cultures or countries has not been validated in the current research. People from different cultures may have different reactions and behaviors in response to stimuli. For instance, people from a collectivist culture may attach more importance to social relationships than those from an individualist culture [59]. Future research could collect data from other countries to re-test the current research model.

## 7. Conclusions

In response to the prevalence of misinformation on social media in the age of COVID-19, this study examines the effects of individuals’ social media dependency on their misinformation sharing behavior with a focus on the underlying mechanisms. Building on the framework of S-O-R, an integrated model was developed to clarify the development of misinformation sharing. A total of 393 responses were obtained using a survey method. The results support most of the proposed hypotheses. In theoretical terms, this study uncovers the processes underlying the relationship between social media dependency and individuals’ misinformation sharing. In practical terms, this study provides actionable guidelines on regulating the use of social media during the COVID-19 pandemic. This study also has several limitations that merit future research.

## Figures and Tables

**Table 1:** The details of samples (*n* = 393).

Demographics	Count (%)	Demographics	Count (%)
*Age*		*Education*	
18–25	127 (32.3%)	High school or below	23 (5.9%)
26–30	104 (26.5%)	College	285 (72.5%)
31–40	128 (32.6%)	Graduate school or above	85 (21.6%)
41–50	19 (4.8%)	*Information sharing frequency*	
More than 50	15 (3.8%)	Less than 3 times	27 (6.9%)
*Gender*		3–10 times	123 (31.3%)
Female	225 (57.3%)	11–20 times	97 (24.7%)
Male	168 (42.7%)	More than 20 times	146 (37.1%)
*Usage Experience*		*Working status*	
Less than 6 months	5 (1.3%)	Employed full time	262 (66.7%)
6 months–1 year	10 (2.5%)	Student	90 (22.9%)
1–3 years	55 (14%)	Self-employed	31 (7.9%)
4–6 years	163 (41.5%)	Unemployed or retired	6 (1.5%)
7 years and above	160 (40.7%)	Others	4 (1%)

**Table 2:** Standard item loadings.

Construct	Items	Mean	STD	Loading	CR	AVE
Informational dependency (ID)	ID1	6.03	0.96	0.85	0.88	0.71
	ID2	5.67	1.11	0.81		
	ID3	5.90	1.02	0.86		
Social dependency (SD)	SD1	5.97	1.15	0.80	0.90	0.64
	SD2	5.77	1.22	0.80		
	SD3	5.66	1.17	0.76		
	SD4	5.76	1.32	0.86		
	SD5	5.36	1.38	0.76		
Perceived information timeliness (PIT)	PIT1	6.13	1.02	0.83	0.87	0.69
	PIT2	5.75	1.07	0.82		
	PIT3	5.96	1.00	0.85		
Perceived socialization (PS)	PS1	6.13	0.96	0.74	0.82	0.52
	PS2	5.12	1.28	0.73		
	PS3	5.64	1.04	0.70		
	PS4	5.28	1.17	0.74		
Information overload (IO)	IO1	4.58	1.33	0.88	0.76	0.80
	IO2	4.03	1.55	0.91		
	IO3	3.96	1.54	0.82		
Social overload (SO)	SO1	5.83	1.11	0.78	0.88	0.65
	SO2	5.22	1.28	0.78		
	SO3	5.32	1.31	0.82		
	SO4	5.52	1.17	0.84		
Positive affect (PA)	PA1	6.12	0.95	0.83	0.88	0.70
	PA2	5.77	1.03	0.84		
	PA3	5.87	1.06	0.84		
Negative affect (NA)	NA1	5.26	1.30	0.82	0.88	0.65
	NA2	4.64	1.42	0.83		
	NA3	4.75	1.46	0.78		
	NA4	5.09	1.31	0.81		
Misinformation sharing (MIS)	MIS1	5.62	1.02	0.84	0.87	0.69
	MIS2	5.70	1.00	0.81		
	MIS3	5.89	1.00	0.84		

**Table 3:** Correlation matrix.

Construct	ID	SD	PIT	PS	IO	SO	PA	NA	MIS	Age	Gender	Education	Experience
Informational dependency	**0.84**												
Social dependency	0.41	**0.80**											
Perceived information timeliness	0.53	0.28	**0.83**										
Perceived socialization	0.47	0.64	0.52	**0.72**									
Information overload	0.06	0.06	0.06	0.16	**0.87**								
Social overload	0.43	0.61	0.58	0.55	0.16	**0.80**							
Positive affect	0.46	0.44	0.48	0.55	0.07	0.55	**0.84**						
Negative affect	0.27	0.24	0.26	0.36	0.45	0.38	0.32	**0.81**					
Misinformation sharing	0.50	0.48	0.42	0.52	0.12	0.58	0.55	0.37	**0.83**				
Age	0.06	0.12	0.01	0.07	-0.22	0.16	0.08	-0.15	0.06	**1.00**			
Gender	0.10	0.02	0.05	0.05	0.05	-0.02	-0.02	0.20	0.02	-0.20	**1.00**		
Education	0.12	0.10	0.15	0.15	0.17	-0.06	-0.02	0.16	0.06	-0.29	0.15	**1.00**	
Usage experience	0.21	0.23	0.16	0.18	0.01	0.08	0.13	0.11	0.14	0.10	0.02	0.15	**1.00**

Note: The diagonal figures (in bold) show the square roots of AVE.

**Table 4:** Mediation analysis.

Relationship	Direct Effect without Mediator	Direct Effect with Mediator	Mediation Effect
PIT→PA→MIS	0.147 *	0.084 ^n.s.^	Full mediation
PS→PA→MIS	0.219 **	0.140 *	Partial mediation
IO→NA→MIS	0.003 ^n.s.^	−0.036 ^n.s.^	No mediation
SO→NA→MIS	0.401 ***	0.304 ***	Partial mediation

Notes: *: *p* < 0.05; **: *p* < 0.01; ***: *p* < 0.001; n.s.: not significant.

## Data Availability

The data of this study are available from the corresponding author upon reasonable request.

## Appendix A. Measurement Instruments

**Table A1:** Measures for constructs.

Construct	No.	Item	References
Informational dependency	ID1	I usually get information through social media during COVID-19.	[30]
	ID2	I usually utilize information gained from social media during COVID-19.	
	ID3	I immediately update information received from social media during COVID-19.	
Social dependency	SD1	I consider how to act with friends, relatives, or acquaintances in social media during COVID-19.	[30]
	SD2	I get ideas about how to approach others from social media during COVID-19.	
	SD3	I have something to do with my friends by using social media during COVID-19.	
	SD4	I use social media to have fun with family or friends during COVID-19.	
	SD5	By using social media, I am a part of social events without having to be there during COVID-19.	
Perceived information timeliness	PIT1	The information about COVID-19 on social media is current.	[47]
	PIT2	The information about COVID-19 on social media is timely.	
	PIT3	The information about COVID-19 on social media is up to date.	
Perceived socialization	PS1	I talk about things with others while using social media during COVID-19.	[51]
	PS2	I feel like I belong to a community while using social media during COVID-19.	
	PS3	I meet interesting people while using social media during COVID-19.	
	PS4	I get peer support from others while using social media during COVID-19.	
Information overload	IO1	I am often distracted by the excessive amount of information on social medial about COVID-19.	[2]
	IO2	I find that I am overwhelmed by the amount of information that I process on a daily basis from social media about COVID-19.	
	IO3	I receive too much information regarding the COVID-19 pandemic to form a coherent picture of what’s happening.	
Social overload	SO1	I care too much about my friends’ well-being on social media during COVID-19.	[57]
	SO2	I deal too much with my friends’ problems on social media during COVID-19.	
	SO3	I care for my friends too much on social media during COVID-19.	
	SO4	I pay too much attention to my friends’ posts on social media during COVID-19.	
Positive affect	Participants were asked to rate the intensity of the emotion they experienced in a particular situation during COVID-19: PA1: desire; PA2: relaxation; PA3: happiness.		[39]
Negative affect	Participants were asked to rate the intensity of the emotion they experienced in a particular situation during COVID-19: NA1: sadness; NA2: fear; NA3: anger; NA4: shock.		[40]
Misinformation sharing	MIS1	I have shared content related to the COVID-19 virus that I later found out was a hoax.	[2,36]
	MIS2	I share content on COVID-19 even if sometimes I feel the content may not be correct.	
	MIS3	I share content on social media related to COVID-19 without checking facts through trusted sources.	
All items are using seven-point Likert scale, with 1 = strongly disagree; 7 = strongly agree.			

This questionnaire can be found at: https://www.wjx.cn/vm/exUK4m9.aspx#, accessed on 15 January 2020.
